# Cervical cancer‐specific long non‐coding RNA landscape reveals the favorable prognosis predictive performance of an ion‐channel‐related signature model

**DOI:** 10.1002/cam4.7389

**Published:** 2024-06-12

**Authors:** Bochang Wang, Wei Wang, Wenhao Zhou, Yujie Zhao, Wenxin Liu

**Affiliations:** ^1^ Department of Gynecological Oncology Tianjin Medical University Cancer Institute & Hospital, National Clinical Research Center for Cancer, Tianjin's Clinical Research Center for Cancer, Key Laboratory of Cancer Prevention and Therapy Tianjin China; ^2^ Tianjin Cancer Hospital Airport Hospital, National Clinical Research Center for Cancer Tianjin China; ^3^ Shenzhen Engineering Center for Translational Medicine of Precision Cancer Immunodiagnosis and Therapy YuceBio Technology Co., Ltd. Shenzhen China

**Keywords:** cervical cancer, ion channel, lncRNA, prognostic model, tumor microenvironment

## Abstract

**Background:**

Ion channels play an important role in tumorigenesis and progression of cervical cancer. Multiple long non‐coding RNA genes are widely involved in ion channel‐related signaling regulation. However, the association and potential clinical application of lncRNAs in the prognosis of cervical cancer are still poorly explored.

**Methods:**

Thirteen patients with cervical cancer were enrolled in current study. Whole transcriptome (involving both mRNAs and lncRNAs) sequencing was performed on fresh tumor and adjacent normal tissues that were surgically resected from patients. A comprehensive cervical cancer‐specific lncRNA landscape was obtained by our custom pipeline. Then, a prognostic scoring model of ion‐channel‐related lncRNAs was established by regression algorithms. The performance of the predictive model as well as its association with the clinical characteristics and tumor microenvironment (TME) status were further evaluated.

**Results:**

To comprehensively identify cervical cancer‐specific lncRNAs, we sequenced 26 samples of cervical cancer patients and integrated the transcriptomic results. We built a custom analysis pipeline to improve the accuracy of lncRNA identification and functional annotation and obtained 18,482 novel lncRNAs in cervical cancer. Then, 159 ion channel‐ and tumorigenesis‐related (ICTR‐) lncRNAs were identified. Based on nine ICTR‐lncRNAs, we also established a prognostic scoring model and validated its accuracy and robustness in assessing the prognosis of patients with cervical cancer. Besides, the TME was characterized, and we found that B cells, activated CD8+ T, and tertiary lymphoid structures were significantly associated with ICTR‐lncRNAs signature scores.

**Conclusion:**

We provided a thorough landscape of cervical cancer‐specific lncRNAs. Through integrative analyses, we identified ion‐channel‐related lncRNAs and established a predictive model for assessing the prognosis of patients with cervical cancer. Meanwhile, we characterized its association with TME status. This study improved our knowledge of the prominent roles of lncRNAs in regulating ion channel in cervical cancer.

## 1 INTRODUCTION

As the world's fourth most common female cancer,^1^ cervical cancer (CC) accounts for about 0.60 million new cases and 0.34 million cancer‐related deaths in 2020.^2^ Increasing effective screening and the vaccination of human papillomavirus (HPV), the incidence of CC showed a sharp decline in the United States from 1975 to 2010.^3^ In contrast, approximately 85% and 90% of new cases and deaths of CC occurred in low‐ and middle‐income countries.^4^ This demonstrates that early screening and HPV vaccination are effective strategies in CC prevention. However, focusing on the prognosis of CC patients is still necessary. Currently, alongside traditional treatment methods such as surgery, chemotherapy, and radiation, immunotherapy has emerged as an important treatment strategy for advanced or recurrent patients with CC.^3^ Whereas, the clinical benefits of these treatments remain limited to a subgroup of patients, especially in patients with persistent, recurrent, and metastatic CC.^1^ Therefore, it is crucial to identify an accurate prognostic biomarker to guide CC treatment and improve their prognosis.

Ion channels, a major class of membrane proteins, contain an aqueous pore and are ubiquitously expressed within cells. They regulate multiple biological processes by facilitating the opening of their pores, such as cell proliferation, migration, and apoptosis.^5^ Aberrant ion channels' activity can impair these processes and lead to disease in various cancers.^6^ Recent works revealed that ion channel signaling play important roles in the metastasis and prognosis of CC.^7^, ^8^, ^9^ CLCN3 (Chloride Channel 3) can promote tumor metastasis and is associated with the prognosis of CC.^7^ The overexpression of SCN8A (Nav1.6) can promote tumor invasion of CC.^8^ AQP8 (Aquaporin 8) is involved in the progression of CC.^9^ Therefore, ion‐channel‐related signatures may be an important prognostic indicator of CC.

As a class of molecules that function at the RNA level, long noncoding RNAs (lncRNAs) have attracted much attention in recent years. Despite the lncRNAs cannot encode proteins, they play key roles in cancer‐related processes by regulating target gene expression.^10^, ^11^, ^12^ Research has shown that lncRNA plays a key role in the pathogenesis of chronic pain by acting on specific ion channels.^13^ However, little is known about the role of ion channel‐related lncRNAs in the prognosis and the regulation of tumor microenvironment (TME) of CC.

In this study, we aim to systematically identify novel lncRNAs by using the transcriptomic data (including both lncRNAs and mRNAs) from CC patients and build a prognostic multi‐lncRNA signature of ion‐channel‐related lncRNAs to predict the prognosis of CC patients.

## 2 MATERIALS AND METHODS

### 2.1 Patient enrollment and clinical sample collection

Thirteen patients with cervical cancer were enrolled at Tianjin Medical University Cancer Institute & Hospital. All patients provided written informed consent. This research was conducted in compliance with all relevant ethical regulations and approved by the Research and Ethical Committee of Tianjin Medical University Cancer Institute & Hospital. Clinical samples, including fresh tumor and matched normal tissues, were obtained surgically before therapy. The basic clinical statistics of enrolled patients are shown in Table 1.

**TABLE 1:** Detailed clinical information of our dataset.

Characteristics	Our dataset
Age (years), no (%)	
<60	12 (92.31)
≥60	1 (7.69)
Pathological stage, no (%)	
NA	2 (15.38)
I	6 (46.15)
II	5 (38.46)

### 2.2 RNA extraction and sequencing

Samples from eight of the thirteen patients performed the whole transcriptome sequencing, and the other samples from five patients performed the mRNA sequencing. For whole transcriptome sequencing, total RNA was extracted from each sample by utilizing the total RNA extraction kit (Tiangen, China) according to the manufacturer's instructions. The RNA quality and integrity were evaluated with the Agilent 2100 bioanalyzer. Ribo‐minus transcriptome libraries were constructed using the NEBNext® UltraTM RNA Library Prep Kit for Illumina® (NEB, United States). The libraries were then sequenced on the Illumina NovaSeq 6000 platform under 150‐bp paired‐end (PE) model following standard procedures. For mRNA‐seq, the Total RNA Extractor (Trizol) kit (Sangon, China) was utilized to extracted total RNA. The RNA integrity and quantity were measured with gel electrophoresis, the NanoPhotometer® spectrophotometer (IMPLEN, CA, USA), and the Qubit® 2.0 Fluorometer (Invitrogen). mRNA transcriptome libraries were generated by using VAHTSTM mRNA‐seq V2 Library Prep Kit for Illumina® (Illumina, USA). The ctDNA libraries were then sequenced on the Illumina NovaSeq 6000 platform with 150‐bp PE model following standard procedures.

### 2.3 Public data collection

In this study, 119 raw transcriptome data from CC patients, including 107 tissue samples and 12 peripheral blood samples, were obtained from the Gene Expression Omnibus.^14^, ^15^, ^16^ The statistic of public datasets used in this study are shown in Table S1. The gene expression data and corresponding clinical information of CC patients were downloaded from The Cancer Genome Atlas database (TCGA, https://xenabrowser.net or https://portal.gdc.cancer.gov). A total of 309 transcriptome data were downloaded. To calculate the correlation between lncRNA expression and the prognosis of CC patients, 3 normal samples, 2 metastasis samples, and 13 samples without survival information were removed. Then, 291 CC patients were selected and used for downstream analysis, which were further randomly classified into a training cohort (*n* = 232) and testing cohort 1 (*n* = 59) at a 4:1 ratio for further analysis.^17^, ^18^ Genes whose total expression amounts were less than 10 were removed, and then log2 transformation was performed. In addition, one of the GEO datasets (GSE168009) containing the data of patients treated with concurrent chemoradiotherapy was utilized as independent testing cohort 2.

### 2.4 Identification of novel lncRNAs

For raw tissue transcriptome data, TrimGalore‐0.6.0 was adopted to remove sequencing adapters and low‐quality reads with the parameter ‘–quality 30’. Then clean reads were mapped to the human reference genome (the UCSC hg38/GRCh38 version) using STAR v.2.7.8a^19^ with the parameter ‘–twopassMode Basic’. Next, StringTie v2.1.6^20^ was utilized to de novo assemble the transcripts in each sample with default parameters.

To identify novel lncRNAs, we first used the cuffmerge and cuffcompare functions (Cufflinks v2.2.1)^21^ to merge transcripts and compare them with protein‐coding genes (GENCODE v38)^22^ and ReflncRNA genes,^23^ respectively. Subsequently, based on the results outputted by the cuffcompare function, the transcripts identified as ‘i, u, x’ were reserved. In length and exon filtering, the transcripts with length less than 200 nt and exon numbers less than 2 were removed. In addition, the transcripts assembled in less than two samples were also removed. In coding ability filtering, transcripts identified as ‘coding’ by Coding Noncoding Index (CNCI)^24^ or Coding Potential Calculator 2 (CPC2)^25^ were removed. Finally, the remaining lncRNAs were regarded as CC‐specific novel lncRNAs.

### 2.5 Identification and functional prediction of Ion‐channel‐ and tumorigenesis‐related (ICTR‐) lncRNAs

Transcripts per million (TPM) values and reads counts of each raw tissue transcriptome data were quantified by Kallisto v.0.46.2.^26^ Genes whose total expression amounts were less than 5 and expressed in less than two samples were removed. TR‐lncRNAs and TR‐coding genes were obtained by comparing tumor and normal samples, where *p* value <0.05 and |log2 expression fold change| >1 served as the cutoffs by DESeq2 (R package).^27^ The intersection analysis was exploited to further identify the relative reliability of TR‐lncRNAs and TR‐mRNAs between the two datasets, respectively. The KOBAS 3.0 (http://kobas.cbi.pku.edu.cn) was used to analyze the Kyoto Encyclopedia of Genes and Genomes (KEGG) pathway enrichment^28^ and to annotate the functions of TR‐mRNAs, where *p* value <0.05 served as the cutoffs.

Ion‐channel genes were downloaded from the HUGO Gene Nomenclature Committee (HGNC) database. Pearson correlation analysis was conducted to determine ion‐channel‐related TR (ICTR)‐lncRNAs, where adjusted *p* value <0.05 and absolute value of the correlation coefficient more than 0.4 served as the cutoff values. Further, the intersection of ICTR‐lncRNAs and the genes expressed in TCGA data was selected for further analysis.

Next, using TPM values of ICTR‐lncRNAs and all mRNAs across samples, we predicted the ICTR‐lncRNA functions based on the co‐expression networks. Specifically, *p* values of Pearson correlation coefficients for ICTR‐lncRNA and mRNA pairs were determined by the WGCNA method. Then, the values were adjusted using the Bonferroni multiple test correction method. The gene pairs that meet the above conditions (adjusted *p* value <0.05 and absolute value of the correlation coefficient greater than 0.4) were added in the co‐expression network. The functions of ICTR‐lncRNAs were annotated by adopting hub‐based methods, for which the functions of ICTR‐lncRNAs were assigned according to the enrichment results of their connected mRNA genes.

### 2.6 Generation of prognostic scoring model based on ICTR‐lncRNAs

To screen candidate ICTR‐lncRNAs that were highly related to the prognosis of CC patients, Univariate Cox regression analysis was performed. ICTR‐lncRNAs with a *p*‐value <0.05 calculated by the univariate Cox regression were enrolled into the least absolute shrinkage and selection operator (LASSO) regression model for further filtering the prognosis‐associated ICTR‐lncRNAs to avoid overfitting. The ICTR‐lncRNAs whose coefficients are non‐zero in the LASSO model were retained. Further, the multivariable Cox regression was adopted to select the final ICTR‐lncRNAs included in the prognostic scoring model. The ICTR‐lncRNA score of each patient was obtained by the expression levels of lncRNAs multiplied by a regression coefficient from multivariable Cox regression output. The formula is as follows:

$$\text{Risk score}=\sum_{n=1}^{n}\left({\text{Coefficient}}_{\text{lncRNA}\;i}\times {\text{Expression level}}_{\text{lncRNA}\;i}\right)$$

All patients' ICTR‐lncRNA scores were determined based on the formula above. According to the median risk score, the patients were then classified into high‐risk and low‐risk groups. Kaplan–Meier (K‐M) analysis was utilized to compare the differences in overall survival (OS). Receiver‐operating characteristic curves (ROCs) analysis was performed to estimate the prognostic capacity of the model.

To further validate the accuracy and robustness of our prognostic model, we conducted the K‐M and ROC analyses in testing cohort 1 and the entire cohort. Due to the lack of survival information for cohort 2, we only used ROC analysis to verify the predictive efficacy in predicting the treatment response. In addition, the associations of the model with clinical characteristics (age, histologic grade, and pathological stage) were also evaluated, and the independence of the ICTR‐lncRNA signature score was assessed.

### 2.7 Development of prognostic nomogram

We developed a nomogram model using the ICTR‐lncRNA signature score and pathological stage to forecast the survival rates of CC patients. Based on the nomogram score system, the 1‐, 3‐, and 5‐year survival of patients were predicted according to the total point for each sample. To evaluate the precision of the nomogram, the concordance index (C‐index) was analyzed in the training or testing cohort 1, as well as the entire cohort.

### 2.8 Tumor microenvironment analysis

To further explore the relationship between ICTR‐lncRNA score and tumor microenvironment status, we first obtained the marker gene set for 28 immune cell types from Charoentong et al.^29^ and the marker gene set for tertiary lymphoid structures (TLS) from Cabrita et al.^30^ Then, the ssGSEA method (“GSVA” package in R) was performed to calculate the levels of immune cell infiltrations and TLS score for each sample.

### 2.9 The signature ICTR‐lncRNA expression patterns in peripheral blood samples

The cuffmerge function was used to merge transcripts from all peripheral blood samples. And then the cuffcompare function was adopted to compare these primary assembled transcripts with mRNAs, ReflncRNA genes, and ICTR‐lncRNAs, respectively. TPM values of ICTR‐lncRNAs in each blood sample were quantified by Kallisto v.0.46.2.^26^

### 2.10 Statistical analysis

Statistical analyses and visualization were performed using R software with version 4.2.2. Univariate and multivariate Cox regression and LASSO (“survival,” “survminer,” and “glmnet” packages in R) were adopted to identify prognostic associated lncRNAs and develop the prognostic model. Fisher's exact test was exploited to compare categorical variables. The differences in the K‐M survival curves and continuous variables between the two groups were performed by Log‐rank tests and the one‐tailed Wilcoxon test, respectively. Pearson correlation analysis was used to determine the association between two continuous variables. Forest plots (“forestplot” package) were used to present the results of univariate and multivariate Cox regression analyses. The “rms” and “survival” packages in R were used to build nomogram model. *p* value <0.05 was considered significant.

## 3 RESULTS

### 3.1 Systematic identification of lncRNAs in CC

**FIGURE 1:**
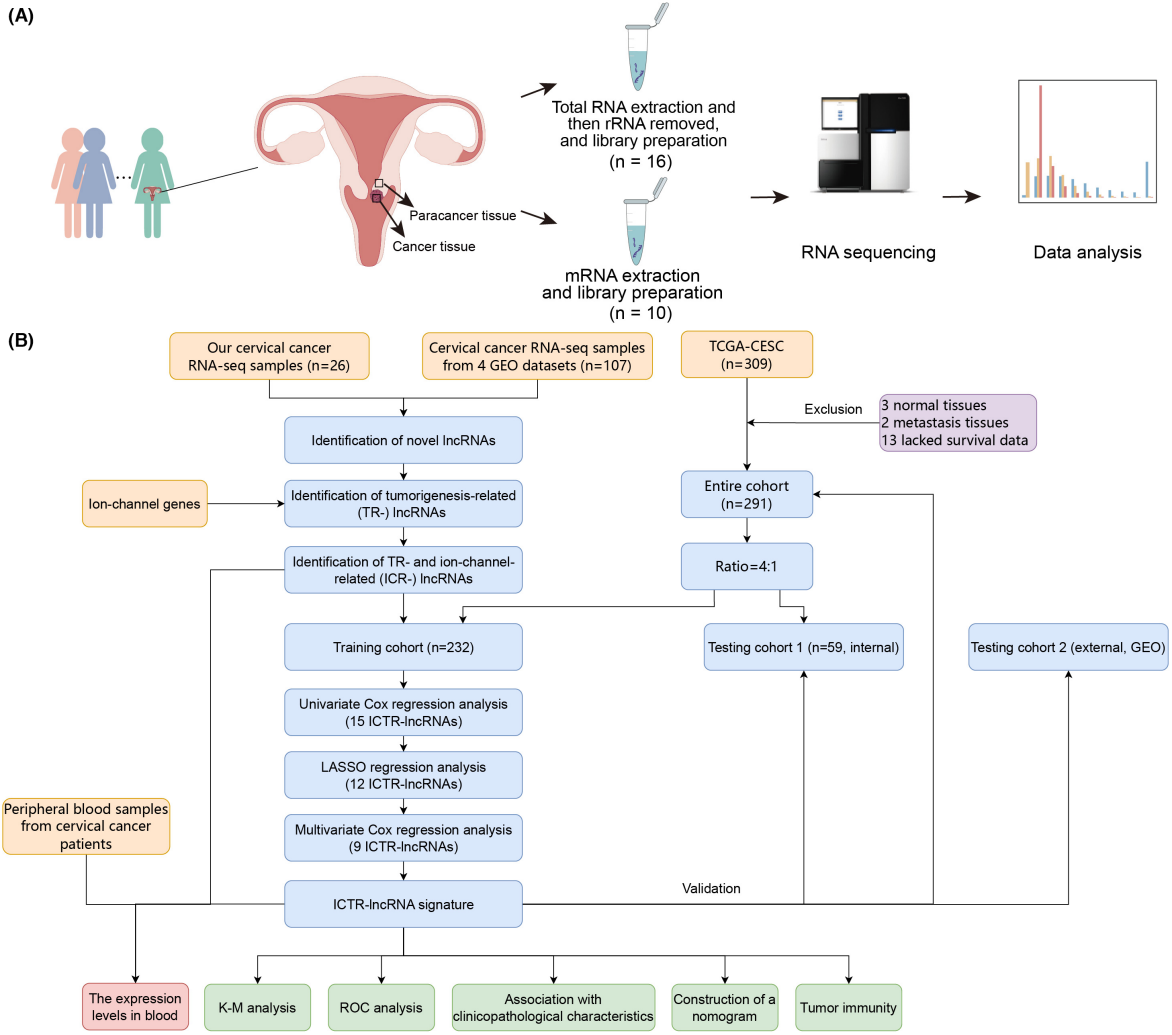
Study design. (A) Overview of clinical sample collection, RNA sequencing, and analysis workflow. (B) Flowchart of the study. ROC analysis, receiver‐operating characteristic analysis; K–M, Kaplan–Meier.

**FIGURE 2:**
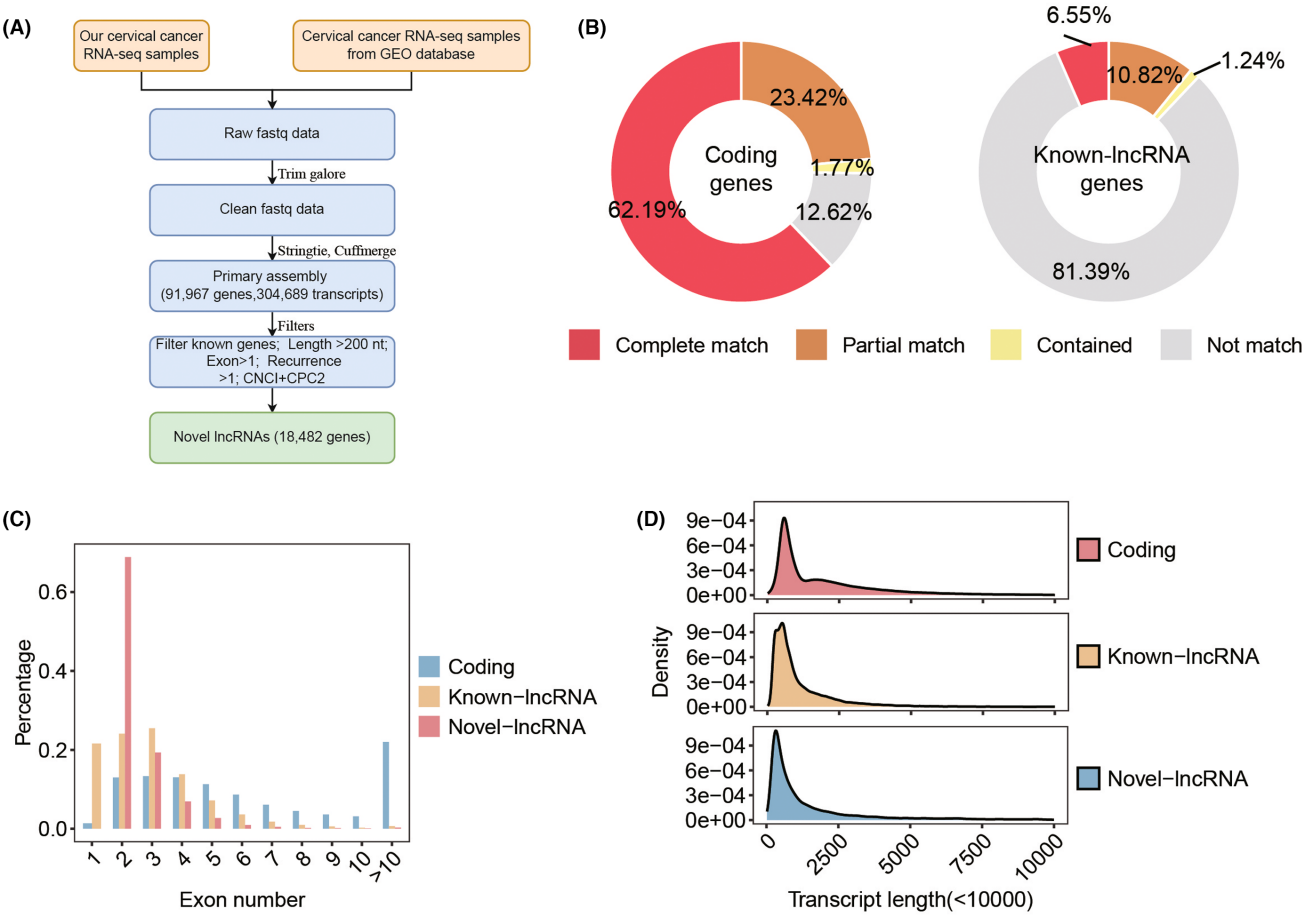
Characteristics of novel identified lncRNA. (A) Flow diagram illustrating the identification process of novel lncRNAs. (B) The comparison of primary assembled transcripts and protein‐coding genes (left) and known lncRNAs (right). (C–D) Characterization of novel lncRNAs in exon number. (C) and transcript length (D).

To identify cervical cancer‐specific lncRNAs, we first collected thirteen pairs of tumor and normal tissue samples for whole transcriptome sequencing (Figure 1A). Subsequently, by integrating these data with raw tissue samples of CC patients collected from the public database, a total of 133 transcriptome data were adopted to identify cervical cancer‐specific lncRNAs. The detailed flowchart of the study is shown in Figure 1B. After clean read alignment and de novo assembly of these tissue data, 91,967 genes were assembled (Figure 2A). By comparing these primary assembled genes with the protein‐coding genes, 87.38% of the coding genes (GENOCODE v38) could be assembled (62.19%, 23.42%, and 1.77% were completely matched, partially matched, and contained, respectively; Figure 2B left). By comparing these primary assembled genes with the known lncRNAs, a lower detection rate than coding genes, only 18.61% of the known lncRNAs could be verified (6.55%, 10.82%, and 1.24% were completely matched, partially matched, and contained, respectively; Figure 2B right). Through our custom pipeline, we finally identified 18,482 novel lncRNAs (Figure 2A). The distribution of exon numbers (Figure 2C) and transcript lengths (Figure 2D) of these lncRNAs were close to known lncRNAs. The number of exons was mainly distributed in the 2–5 range (Figure 2C), and the mean transcript length was 1.4 k nt of novel lncRNAs (Figure 2D).

### 3.2 Identification of ICTR‐lncRNA based on ion channel genes

By comparing tumor and normal tissues, 713 TR‐lncRNAs were identified, 209 of which were novel lncRNAs (Figure S1A and Table S2). Meanwhile, 796 TR‐coding genes were obtained, including 330 upregulated and 466 downregulated genes (Figure S1B and Table S3). KEGG enrichment pathways showed that upregulated genes were mainly enriched in the cell cycle, p53 and chemokine signaling pathways, DNA replication, cytokine‐cytokine receptor interaction, and mismatch repair (Figure S1C, Table S4). Downregulated genes were mainly enriched in the Ras, PI3K‐Akt, and MAPK signaling pathways (Figure S1D and Table S4).

Based on these TR‐lncRNAs and ion‐channel genes, Pearson correlation and intersection analysis analyses were utilized to screen ion‐channel‐related TR‐lncRNAs (ICTR‐lncRNAs), and 159 ICTR‐lncRNAs were obtained for modeling (Table S5).

### 3.3 Establishment of prognostic scoring model

To establish a prognostic scoring model for CC patients, we downloaded expression profiles with corresponding clinical data from the public database. By Fisher's exact test, we found no significant differences in age, pathological stage, histologic grade, or survival event between different groups (all *p* > 0.05; Table S6).

**FIGURE 3:**
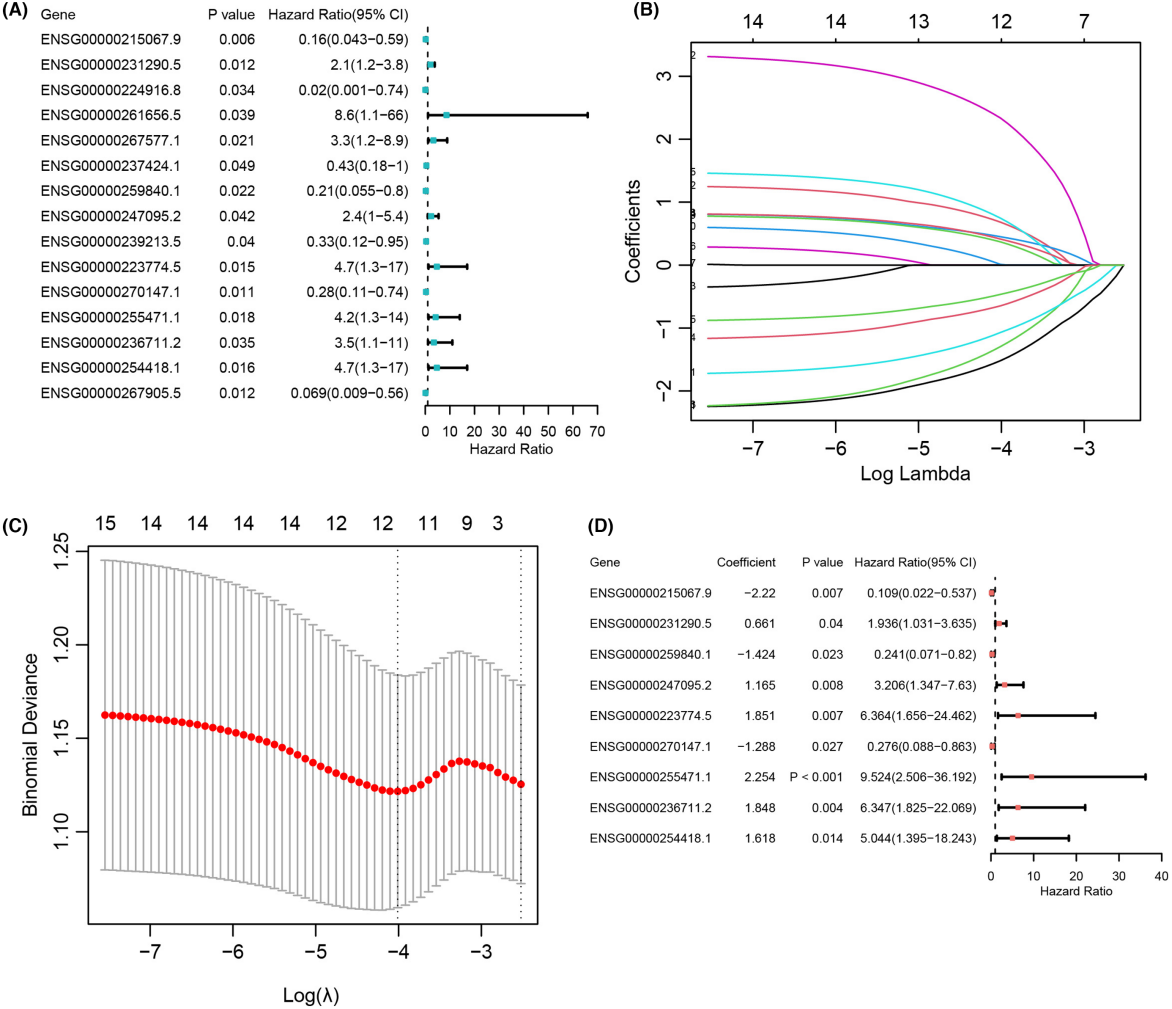
Construct the prognostic model by ICTR‐lncRNAs. (A) Forest plots showed the *p* value, HR, and 95% CI of 15 ICTR‐lncRNAs identified by univariate Cox regression analysis. (B) The distribution plot of the LASSO coefficients. (C) Twelve variables were retained when the partial likelihood deviation reached the minimum (Log Lambda = −4.010). (D) Forest plots showed the coefficient, *p* value, HR, and 95% CI of nine ICTR‐lncRNAs identified by multivariable Cox regression analysis.

Fifteen prognostic‐related ICTR‐lncRNAs were obtained by Univariate Cox regression analysis (*p* < 0.05, Figure 3A), which were further inputted in LASSO. Then, with the log‐transformed lambda was equal to −4.010, we screened 12 ICTR‐lncRNAs (Figure 3B,C). Further, by multivariate Cox regression analysis, a total of nine ICTR‐lncRNAs were retained for constructing the prognostic scoring model (*p* < 0.05; Figure 3D and Table S7). The detailed formula to compute the risk score was shown in Method 2.3.

### 3.4 Assessment and validation of the prognostic scoring model

**FIGURE 4:**
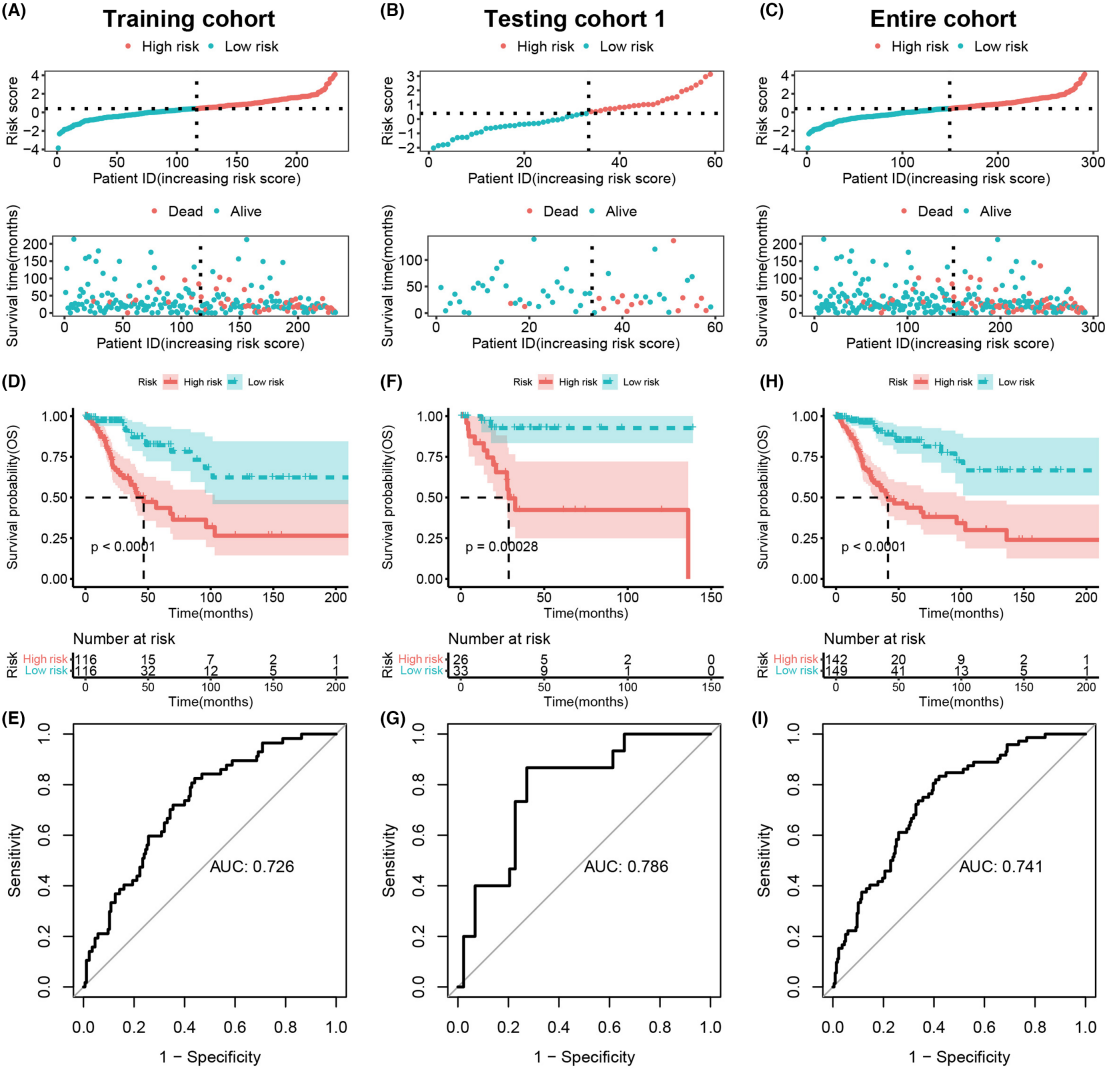
Assessment and authentication of the ICTR‐lncRNA signature in CC. (A–C) The distribution of risk score and overall survival in the training (A), testing 1 (B), and entire cohorts (C). (D–F) K–M analysis revealed that the low‐risk patients exhibited prolonged OS in the training (D), testing 1 (E), and entire cohorts (F). (G–I) ROCs analysis of the ICTR‐lncRNA signature for OS in the training (G), testing 1 (H), and entire cohorts (I).

To evaluate and validate the prognostic scoring model, we first calculated the risk scores of patients in each cohort. In the training cohort, high‐risk and low‐risk groups of CC patients were classified based on the median risk score (Figure 4A). We found that compared with the low‐risk patient group, the high‐risk group showed a higher count of deaths (*p* = 1.427e‐05, Figure 4A). The same results were also reached in testing cohort 1 and the entire cohort (Figure 4B,C). To further clarify the accuracy and robustness of the model for predicting the prognosis of CC patients, KM analysis and log‐rank test were performed. We found a significantly longer OS exhibited in low‐risk patients in the training cohort (*p* < 0.0001, Figure 4D), testing cohort 1 (*p* = 0.00028, Figure 4E), and entire cohort (*p* < 0.0001, Figure 4F). The corresponding AUC values of the ROCs analysis of the model were 0.726, 0.786, and 0.741, respectively (Figure 4G–I). These findings suggested that the model can be considered accurate and robust in the prediction of prognosis in CC patient.

**FIGURE 5:**
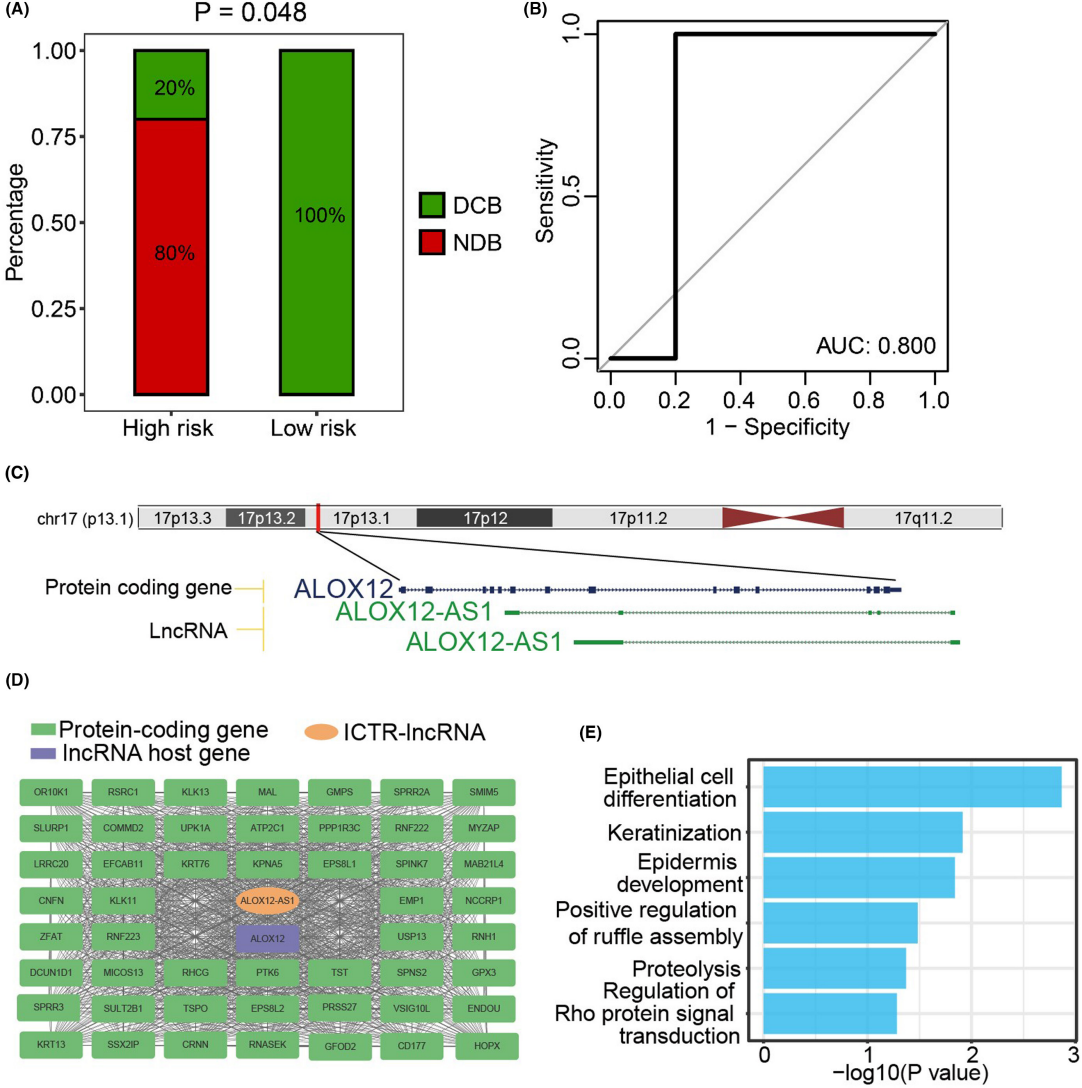
Identification of ICTR‐lncRNA signature for predicting the therapeutic response of CC received concurrent chemoradiotherapy. (A) The concurrent chemoradiotherapy response ratio of ICTR‐lncRNA signature. (B) ROC analysis of ICTR‐lncRNA signature to predict the benefits of concurrent chemoradiotherapy. (C) Genomic view of lncRNA ALOX12‐AS1. (D) The co‐expression network of ALOX12‐AS. Protein‐coding genes co‐expressed with lncRNA genes and the host genes of lncRNA are visually distinguished by the colors green, orange, and purple, respectively. (E) Functional annotations of ALOX12‐AS1 based on co‐expression network.

Moreover, we validated this model in an independent CC dataset (testing cohort 2), which included patients treated with concurrent chemoradiotherapy. Notably, the low‐risk patients showed stronger sensitivity to concurrent chemoradiotherapy (*p* = 0.048, Figure 5A) and the AUC value reached 0.8 (Figure 5B). Collectively, low‐risk patients were more suitable for concurrent chemoradiotherapy.

To gain further insights into the putative functions of ICTR‐lncRNAs in the model, we used hub‐based method by building the coding‐noncoding co‐expression network. According to the functional annotation results, diverse functions of signature ICTR‐lncRNAs were observed, such as cell differentiation, metabolism pathways, and angiogenesis (Figure S3). For example, as the antisense of the *ALOX12* gene, lncRNA *ALOX12‐AS1* was co‐expressed with multiple cancer‐related genes and annotated as epithelial cell differentiation and keratinization, which were complementary to the functions of its host gene (Figure 5C–E).

### 3.5 Association between the prognostic scoring model and clinical features

**FIGURE 6:**
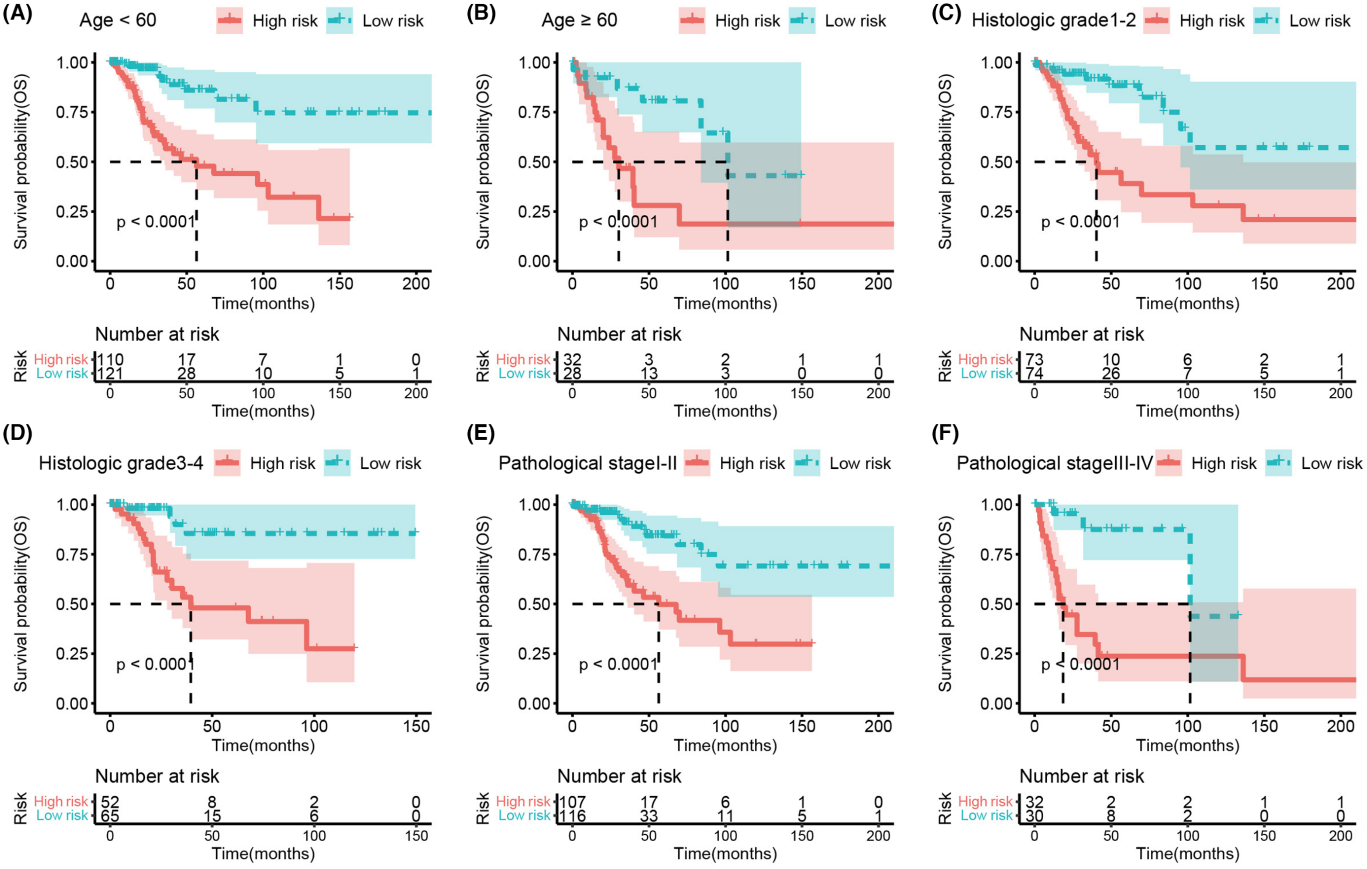
K‐M analysis for the high‐ and low‐risk groups stratified by clinical factors. (A) Age < 60 years. (B) Age ≥ 60 years. (C) Histologic grade 1–2. (D) Histologic grade 3–4. (E) Pathological stage I–II. (F) Pathological stage III–IV.

**FIGURE 7:**
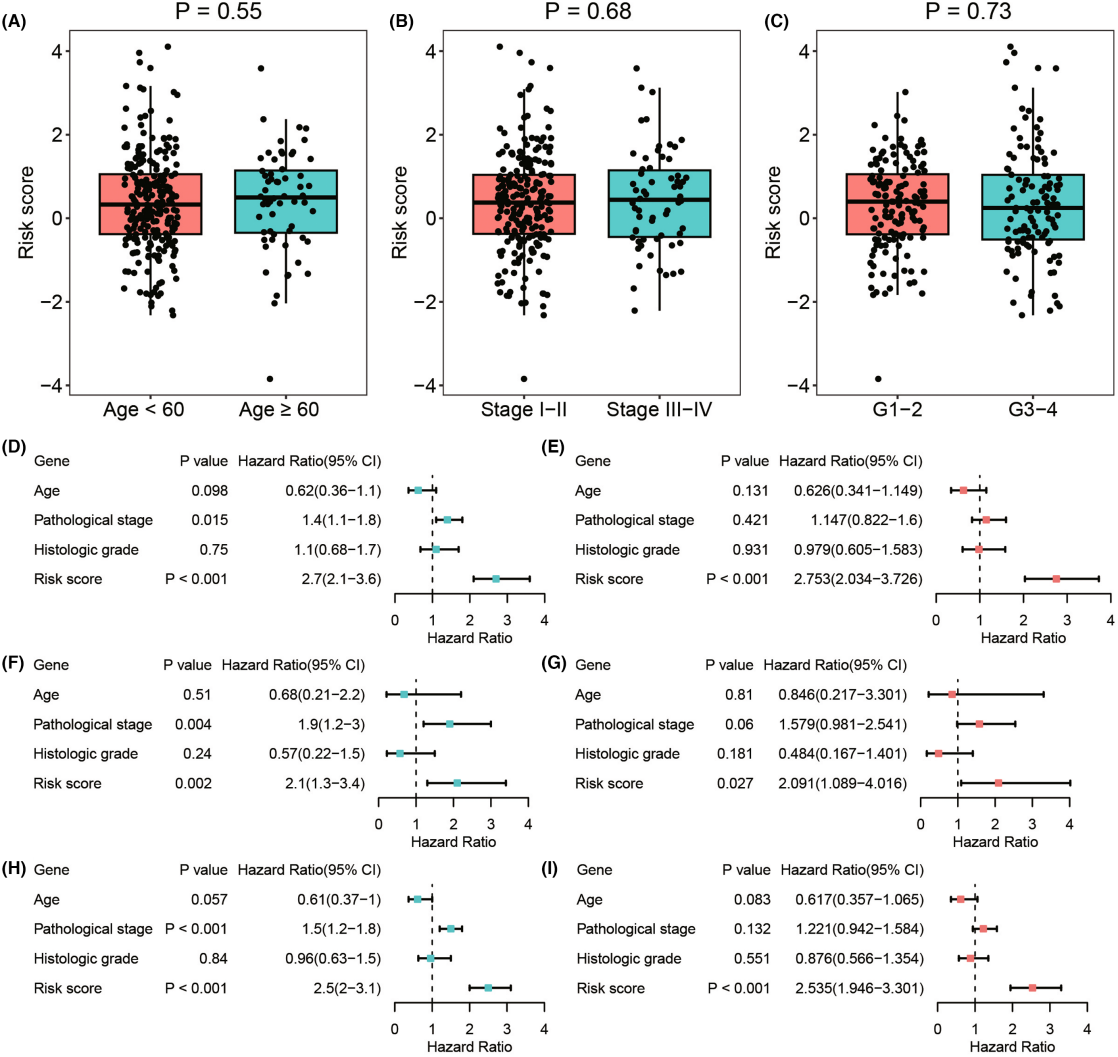
The signature of ICTR‐lncRNA emerged as a standalone prognostic determinant for OS in CC. (A–C) Boxplots showed the distribution of risk scores in age (A), histologic grade (B), and pathological stage (C). (D–I) Univariate and multivariate Cox regression analyses of risk scores and other clinical factors were performed in the training (D, E), testing 1 (F, G), and entire cohorts (H, I).

To investigate the relationship between the predictive model and clinical features, we stratified the patients based on their age (Figure 6A,B), histologic grade (Figure 6C,D), and pathological stage (Figure 6E,F). The results demonstrated that patients in the high‐risk group showed a significant reduction in overall survival (all *p* < 0.0001). Subsequently, we analyzed the difference in risk scores according to these clinical features and found that age, histologic grade, and pathological stage were not significantly related to the risk score (Figure 7A–C).

Next, we investigated whether the ICTR‐lncRNA signature score was an independent predictor for the prognosis of CC patients. The significant differences were observed only in risk scores by univariate (*p* < 0.001, HR = 2.7, 95% CI = [2.1–3.6], Figure 7D) and multivariate Cox regression analyses (*p* < 0.001, HR = 2.753, 95% CI = [2.034–3.726], Figure 7E) in the training cohort. Similar results were found in testing 1 and entire cohorts (Figure 7F–I). These findings indicated that the signature of ICTR‐lncRNAs was an independent prognostic factor for CC.

### 3.6 Survival prediction of CC based on a nomogram model

**FIGURE 8:**
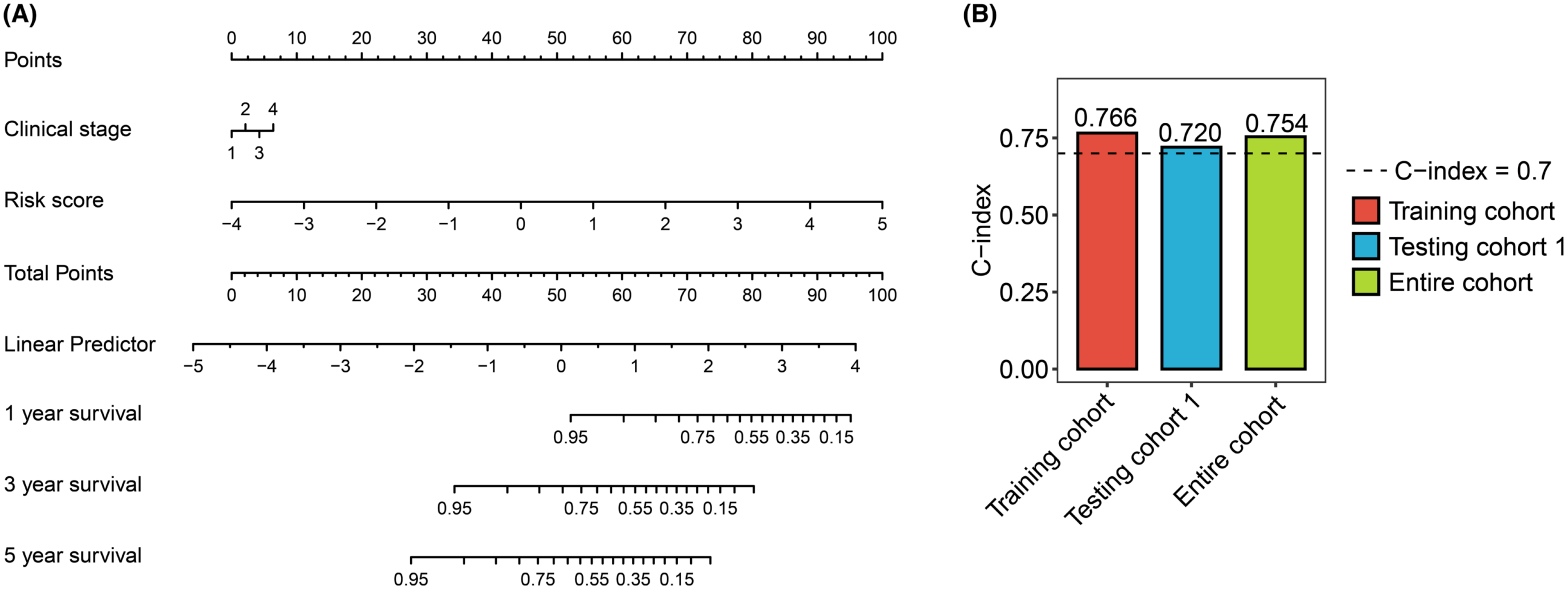
The construction of a nomogram integrating the risk score and pathological stage to forecast the survival duration of patients with CC. (A) The nomogram for estimating the OS of patients with CC at 1, 3, and 5 years. (B) Boxplots showed the C‐index of the nomogram across the training, testing 1, and entire cohorts.

To better predict survival time for patients with CC, a nomogram model to predict 1‐, 3‐, and 5‐year OS was constructed by ICTR‐lncRNA signature and pathological stage (Figure 8A). The results showed that for the training, testing 1, and entire cohorts, the concordance indexes (C‐index) of the nomogram were 0.766, 0.720, and 0.754, respectively (Figure 8B).

### 3.7 The ICTR‐lncRNA signature was correlated with TMEs

**FIGURE 9:**
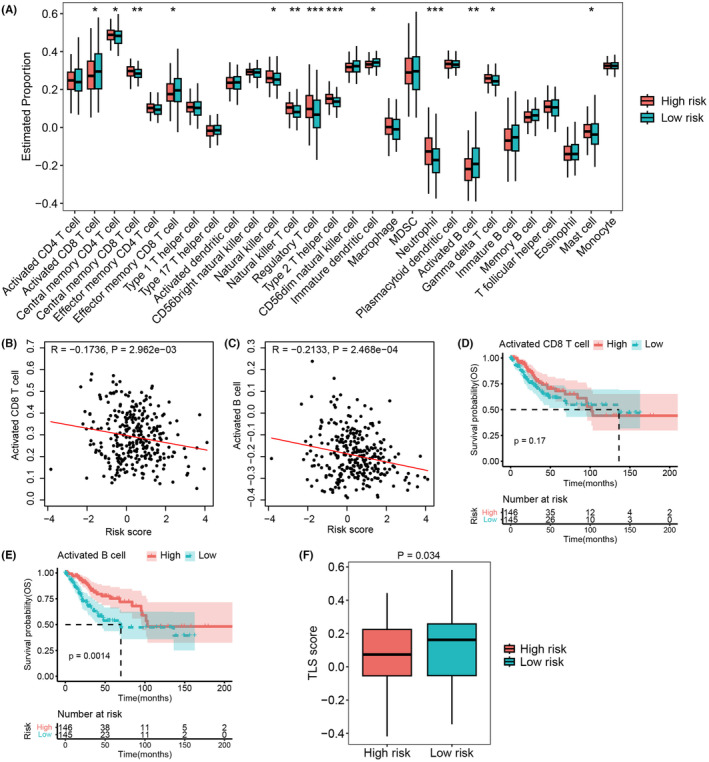
The correlation between the ICTR‐lncRNA signature and immune cell infiltration. (A) The distinction in the infiltration of immune cell types within the high‐ and low‐risk groups as defined by the ICTR‐lncRNA signature. (B, C) The scatter plots displayed the correlation within the risk score and the infiltration of activated CD8 T cell (B) and activated B cell (C). (D, E) K–M analysis showed the OS of activated CD8 T cell (D) and activated B cell infiltration (E). (F) The distinction in TLS scores within the high‐ and low‐risk groups identified by the ICTR‐lncRNA signature. Wilcoxon’s test was used for comparison (^*^p < 0.05, ^**^p < 0.01, ^***^p < 0.001).

Recent works have highlighted the significance of ion channels in the regulation of immune cell activity,^31^, ^32^ so we next explored the association between the ICTR‐lncRNA signature and tumor immunity. The ssGSEA method was utilized to calculate the infiltration levels of various immune cell types based on RNA‐seq data (Figure S2). In comparison with the low‐risk patients, the patients in high‐risk group showed a higher infiltration level in central memory CD4 and CD8 T cell, natural killer cell, natural killer T cell, Treg cell, Th2 cell, neutrophil, gamma delta T cell, and mast cell, whereas a lower degree of infiltration in activated and effector memory CD8 T cell, immature dendritic cell, and activated B cell (all *p* < 0.05, Figure 9A). Recent studies have revealed that CD8+ T and CD20+ B cells, as components of tertiary lymphoid structures (TLS),^30^ were associated with improved survival in most cancers.^30^, ^33^ Interestingly, the low‐risk group showed a higher infiltration level both in activated CD8 T cells and activated B cells (Figure 9A). Furthermore, we found that the risk score was negatively associated with the levels of activated CD8 T (R = −0.1736, *p* = 2.962e‐03, Figure 9B) and activated B cells (R = −0.2133, *p* = 2.468e‐04, Figure 9C). KM analysis demonstrated that the highly infiltration of activated B cell was associated with longer OS, but such trend was not observed in the activated CD8 T cell (Figure 9D,E). In addition, the low‐risk group showed a higher TLS score than the high‐risk group (*p* = 0.034, Figure 9F). These findings suggested that activated CD8+ T and CD20+ B cells as well as TLS were significantly associated with ICTR‐lncRNA signature scores.

### 3.8 The expression of tumor ICTR‐lncRNAs in peripheral blood samples

**FIGURE 10:**
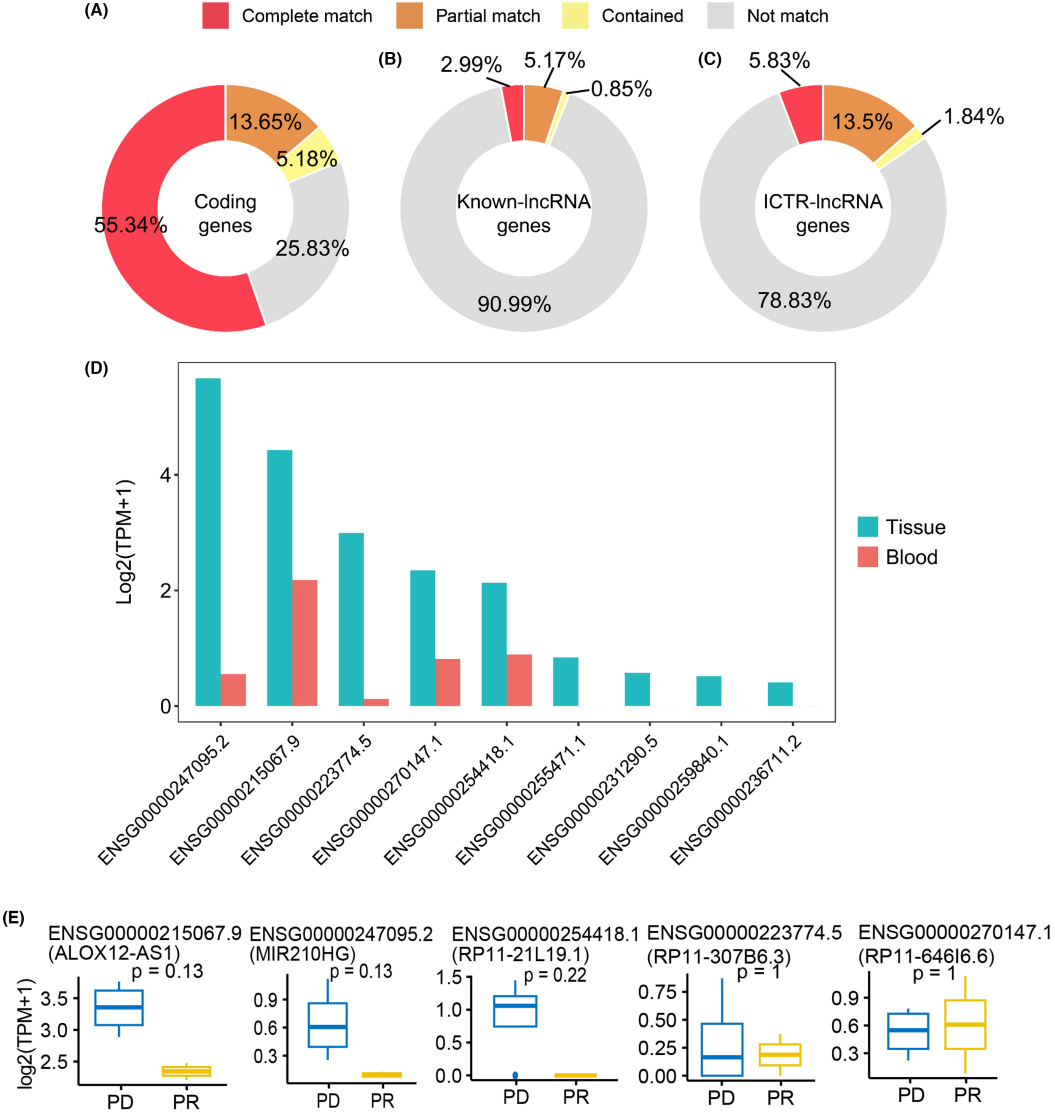
Assessment of ICTR‐lncRNAs expressed in blood samples from CC patients. (A–C) The comparison of primary assembled transcripts in blood samples and protein‐coding genes (A), known lncRNA genes (B), and ICTR‐lncRNAs (C). (D) Bar plots represented the expression levels (log2 TPM) of ICTR‐lncRNAs in the risk model in tissue and blood samples. (E) The comparison of the ICTR‐lncRNAs between the PR and PD group of patients in peripheral blood at baseline. PR, partial response; PD, progressive disease.

Finally, we examined the expression pattern of tumor ICTR‐lncRNAs in peripheral blood from CC patients. After the de novo assembly in blood raw transcriptome data, a total of 31,038 genes were successfully identified, which were then used to compare with protein‐coding genes, known lncRNA genes, and ICTR‐lncRNAs. About 74%, 9%, and 21% of protein‐coding, known lncRNA, and ICTR‐lncRNAs were assembled in blood samples (Figure 10A–C), respectively. Further through the quantitative analysis of gene expression, we found 5/9 ICTR‐lncRNAs in the risk model were detected and showed low expression levels in blood samples (Figure 10D). Moreover, compared with the progressive disease (PD) group of patients, 3 ICTR‐lncRNAs in the partial response (PR) group showed a decreased trend in peripheral blood at baseline (Figure 10E). The results revealed that despite the low expression of lncRNA in blood, the detection of these ICTR‐lncRNAs may provide a new strategy in the development of prognostic markers of CC.

## 4 DISCUSSION

In this work, 436 transcriptome data from CC patients were obtained from our dataset and public database. Based on these data, we performed a comprehensive bioinformatics study to identify cervical cancer‐specific lncRNAs and ICTR‐lncRNAs and construct a prognostic scoring model to predict the prognosis of CC patients. In addition, we also analyzed the association of the model with clinical characteristics and tumor microenvironment status.

By modeling, nine ICTR‐lncRNAs associated with CC prognosis were identified, including ALOX12‐AS1, APCDD1L‐AS1, LA16c‐380A1.1, MIR210HG, RP11‐307B6.3, RP11‐646I6.6, RP11‐736 K20.5, SMAD9‐IT1, and RP11‐21 L19.1. All these genes except RP11‐646I6.6 were downregulated. Through a systematic review of previous studies, we found that the role of these ICTR‐lncRNAs in CC is still poorly explored. *APCDD1L‐AS1* located in 20q13.32. Previous studies have revealed that the abnormal expression of *APCDD1L‐AS1* was related to the tumor growth and metastasis, prognosis, and drug resistance in clear cell renal cell carcinoma, lung adenocarcinoma and squamous cell carcinoma, and oral squamous cell carcinoma.^34^, ^35^, ^36^, ^37^ *MIR210HG* is located at 11p15.5 which is associated with tumor cell proliferation and metastasis in endometrial cancer, breast cancer, cervical cancer, NSCLC, and gastric cancer.^38^, ^39^, ^40^, ^41^, ^42^, ^43^, ^44^, ^45^ RP11‐21 L19.1 located in 11p15.2, and the low expression of which was significantly related with a poor prognosis in kidney renal papillary cell carcinoma.^46^ The functions of the other ICTR‐lncRNAs need to be further explored.

As known to all, TMEs play crucial roles in tumorigenesis, progression, metastasis, and response to therapies.^47^, ^48^ Studies have shown that ion channel genes play essential roles in regulating lymphocyte development and modulating immune response.^31^, ^32^ Therefore, we explored the association of the model with TMEs. Interestingly, we found that activated T and B cells as well as TLS were significantly associated with ICTR‐lncRNAs signature scores. TLS is generally composed of CD8+ T cells and CD20+ B cells,^30^ which are often associated with a good prognostic outcome in most cancers.^30^, ^33^ Our results indicated that the longer survival time of low‐risk patients may be related to the formation of TLS. However, studies on TLS in CC are still fewer and need to receive increasing attention.

Another important finding of our study was that the model can guide the treatment approach in advanced CC patients. The low‐risk patients displayed stronger sensitivity to concurrent chemoradiotherapy, and the AUC value achieved 0.8. These results indicate that the prognostic scoring model may help predict the therapeutic response of patients with CC treated with concurrent chemoradiotherapy.

This study still had some limitations. Firstly, molecular experimental validation of the prognostic scoring model is required to verify our results. In addition, the mechanism of key ion‐channel pathways regulated by ICTR‐lncRNAs remains to be further explored. Moreover, although we have employed multiple datasets that contained both lncRNA data and clinical outcomes from our own generated and publicly available data to perform the analysis, the robustness of our findings still requires more data sets to be validated in terms of survival.

## 5 CONCLUSION

In conclusion, we comprehensively identified cervical cancer‐specific lncRNAs, which extended the current lncRNA catalog and provided help for other researchers to analyze the cervical cancer‐specific lncRNA transcriptome. Through integrative analyses, we identified ion channel‐related lncRNAs and established a prognostic scoring model to predict the prognosis of cervical cancer patients. Meanwhile, we characterized its association with clinical characteristics and tumor microenvironment status. This study improved our understanding of the prominent roles of lncRNAs in regulating ion‐channel in cervical cancer.

## AUTHOR CONTRIBUTIONS

**Bochang Wang:** Formal analysis (equal); investigation (lead); writing – original draft (equal). **Wei Wang:** Formal analysis (equal); visualization (lead); writing – original draft (equal). **Wenhao Zhou:** Formal analysis (equal); visualization (equal). **Yujie Zhao:** Data curation (lead); writing – original draft (supporting). **Wenxin Liu:** Conceptualization (lead); funding acquisition (lead); supervision (lead); writing – review and editing (lead).

## FUNDING INFORMATION

This study was supported by the Foundation of Tianjin Municipal Education Commission of China [grant number 2019ZD033].

## CONFLICT OF INTEREST STATEMENT

The authors declare that the research was conducted in the absence of any commercial or financial relationships that could be construed as a potential conflict of interest.

## ETHICS STATEMENT

This study was performed in accordance with the Declaration of Helsinki and was approved by the Ethics Committee of Tianjin Medical University Cancer Institute & Hospital. Written informed consent was obtained from all patients involved in the study.

## Supporting information


Figures S1–S3.

Tables S1–S8.

## Data Availability

The raw data supporting the conclusions of this article will be made available by the authors, without undue reservation. The other public datasets generated and/or analyzed during the current study are available in the GEO repository (GSE206224, GSE87410, GSE151419, GSE168009, GSE205247, and GSE188305) and TCGA database (https://xenabrowser.net or https://portal.gdc.cancer.gov).

## References

[1] Cohen PA , Jhingran A , Oaknin A , Denny L . Cervical cancer. Lancet. 2019;393(10167):169‐182. doi:10.1016/S0140-6736(18)32470-X 30638582

[2] Sung H , Ferlay J , Siegel RL , et al. Global cancer statistics 2020: GLOBOCAN estimates of incidence and mortality worldwide for 36 cancers in 185 countries. CA Cancer J Clin. 2021;71(3):209‐249. doi:10.3322/CAAC.21660 33538338

[3] Podwika SE , Duska LR . Top advances of the year: cervical cancer. Cancer. 2023;129(5):657‐663. doi:10.1002/CNCR.34617 36609769 PMC10107116

[4] Bhatla N , Aoki D , Sharma DN , Sankaranarayanan R . Cancer of the cervix uteri: 2021 update. Int J Gynaecol Obstet. 2021;155(Suppl 1):28‐44. doi:10.1002/IJGO.13865 34669203 PMC9298213

[5] Prevarskaya N , Skryma R , Shuba Y . Ion channels in cancer: are cancer hallmarks Oncochannelopathies? Physiol Rev. 2018;98(2):559‐621. doi:10.1152/PHYSREV.00044.2016 29412049

[6] Xia J , Wang H , Li S , et al. Ion channels or aquaporins as novel molecular targets in gastric cancer. Mol Cancer. 2017;16(1):54. doi:10.1186/S12943-017-0622-Y 28264681 PMC5338097

[7] Xu B , Jin X , Min L , et al. Chloride channel‐3 promotes tumor metastasis by regulating membrane ruffling and is associated with poor survival. Oncotarget. 2015;6(4):2434‐2450. doi:10.18632/ONCOTARGET.2966 25537517 PMC4385862

[8] Hernandez‐Plata E , Ortiz CS , Marquina‐Castillo B , et al. Overexpression of NaV 1.6 channels is associated with the invasion capacity of human cervical cancer. Int J Cancer. 2012;130(9):2013‐2023. doi:10.1002/IJC.26210 21630263

[9] Shi YH , Rehemu N , Ma H , Tuokan T , Chen R , Suzuke L . Increased migration and local invasion potential of SiHa cervical cancer cells expressing aquaporin 8. Asian Pac J Cancer Prev. 2013;14(3):1825‐1828. doi:10.7314/APJCP.2013.14.3.1825 23679281

[10] Peng WX , Koirala P , Mo YY . LncRNA‐mediated regulation of cell signaling in cancer. Oncogene. 2017;36(41):5661‐5667. doi:10.1038/onc.2017.184 28604750 PMC6450570

[11] Bao S , Zhao H , Yuan J , et al. Computational identification of mutator‐derived lncRNA signatures of genome instability for improving the clinical outcome of cancers: a case study in breast cancer. Brief Bioinform. 2020;21(5):1742‐1755. doi:10.1093/BIB/BBZ118 31665214

[12] Jiang Y , Ye Y , Huang Y , et al. Identification and validation of a novel anoikis‐related long non‐coding RNA signature for pancreatic adenocarcinoma to predict the prognosis and immune response. J Cancer Res Clin Oncol. 2023;149(16):15069‐15083. doi:10.1007/s00432-023-05285-x 37620430 PMC11797502

[13] Felix R , Muñoz‐Herrera D , Corzo‐López A , et al. Ion channel long non‐coding RNAs in neuropathic pain. Pflügers Archiv. European Journal of Physiology. 2022;474(4):457‐468. doi:10.1007/S00424-022-02675-X 35235008

[14] Liu L , Zhao Q , Cheng C , et al. Analysis of bulk RNA sequencing data reveals novel transcription factors associated with immune infiltration among multiple cancers. Front Immunol. 2021;12:644350. doi:10.3389/FIMMU.2021.644350 34489925 PMC8417605

[15] Ruiz FJ , Inkman M , Rashmi R , et al. HPV transcript expression affects cervical cancer response to chemoradiation. JCI Insight. 2021;6(16):138734. doi:10.1172/JCI.INSIGHT.138734 PMC8409981 34255749

[16] Liu X , Zhang X , Liu C , Mu W , Peng J , Song K . Immune and inflammation: related factor alterations as biomarkers for predicting prognosis and responsiveness to PD‐1 monoclonal antibodies in cervical cancer. Discover. Oncology. 2022;13(1):96. doi:10.1007/S12672-022-00560-8 PMC9519820 36171464

[17] Wang M , Perucho JAU , Hu Y , et al. Computed tomographic radiomics in differentiating histologic subtypes of epithelial ovarian carcinoma. JAMA Netw Open. 2022;5(12):e2245141. doi:10.1001/jamanetworkopen.2022.45141 36469315 PMC9855300

[18] Wang J , Yu S , Chen G , et al. A novel prognostic signature of immune‐related genes for patients with colorectal cancer. J Cell Mol Med. 2020;24(15):8491‐8504. doi:10.1111/jcmm.15443 32564470 PMC7412433

[19] Dobin A , Davis CA , Schlesinger F , et al. STAR: ultrafast universal RNA‐seq aligner. Bioinformatics. 2013;29(1):15‐21. doi:10.1093/BIOINFORMATICS/BTS635 23104886 PMC3530905

[20] Pertea M , Pertea GM , Antonescu CM , Chang TC , Mendell JT , Salzberg SL . StringTie enables improved reconstruction of a transcriptome from RNA‐seq reads. Nat Biotechnol. 2015;33(3):290‐295. doi:10.1038/NBT.3122 25690850 PMC4643835

[21] Trapnell C , Williams BA , Pertea G , et al. Transcript assembly and quantification by RNA‐seq reveals unannotated transcripts and isoform switching during cell differentiation. Nat Biotechnol. 2010;28(5):511‐515. doi:10.1038/NBT.1621 20436464 PMC3146043

[22] Frankish A , Diekhans M , Ferreira AM , et al. GENCODE reference annotation for the human and mouse genomes. Nucleic Acids Res. 2019;47(D1):D766‐D773. doi:10.1093/nar/gky955 30357393 PMC6323946

[23] Jiang S , Cheng SJ , Ren LC , et al. An expanded landscape of human long noncoding RNA. Nucleic Acids Res. 2019;47(15):7842‐7856. doi:10.1093/NAR/GKZ621 31350901 PMC6735957

[24] Sun L , Luo H , Bu D , et al. Utilizing sequence intrinsic composition to classify protein‐coding and long non‐coding transcripts. Nucleic Acids Res. 2013;41(17):e166. doi:10.1093/NAR/GKT646 23892401 PMC3783192

[25] Kang YJ , Yang DC , Kong L , et al. CPC2: a fast and accurate coding potential calculator based on sequence intrinsic features. Nucleic Acids Res. 2017;45(W1):W12‐W16. doi:10.1093/NAR/GKX428 28521017 PMC5793834

[26] Bray NL , Pimentel H , Melsted P , Pachter L . Near‐optimal probabilistic RNA‐seq quantification. Nat Biotechnol. 2016;34(5):525‐527. doi:10.1038/nbt.3519 27043002

[27] Love MI , Huber W , Anders S . Moderated estimation of fold change and dispersion for RNA‐seq data with DESeq2. Genome Biol. 2014;15(12):550. doi:10.1186/S13059-014-0550-8 25516281 PMC4302049

[28] Bu D , Luo H , Huo P , et al. KOBAS‐i: intelligent prioritization and exploratory visualization of biological functions for gene enrichment analysis. Nucleic Acids Res. 2021;49(W1):W317‐W325. doi:10.1093/NAR/GKAB447 34086934 PMC8265193

[29] Charoentong P , Finotello F , Angelova M , et al. Pan‐cancer immunogenomic analyses reveal genotype‐immunophenotype relationships and predictors of response to checkpoint blockade. Cell Rep. 2017;18(1):248‐262. doi:10.1016/j.celrep.2016.12.019 28052254

[30] Cabrita R , Lauss M , Sanna A , et al. Tertiary lymphoid structures improve immunotherapy and survival in melanoma. Nature. 2020;577(7791):561‐565. doi:10.1038/S41586-019-1914-8 31942071

[31] Feske S , Wulff H , Skolnik EY . Ion channels in innate and adaptive immunity. Annu Rev Immunol. 2015;33:291‐353. doi:10.1146/annurev-immunol-032414-112212 25861976 PMC4822408

[32] Hamza A , Amit J , Elizabeth LE , et al. Ion channel mediated mechanotransduction in immune cells. Curr Opin Solid State Mater Sci. 2021;25(6):100951. doi:10.1016/j.cossms.2021.100951 35645593 PMC9131931

[33] Munoz‐Erazo L , Rhodes JL , Marion VC , Kemp RA . Tertiary lymphoid structures in cancer ‐ considerations for patient prognosis. Cell Mol Immunol. 2020;17(6):570‐575. doi:10.1038/S41423-020-0457-0 32415259 PMC7264315

[34] Yang W , Zhou J , Zhang Z , et al. Downregulation of lncRNA APCDD1L‐AS1 due to DNA hypermethylation and loss of VHL protein expression promotes the progression of clear cell renal cell carcinoma. Int J Biol Sci. 2022;18(6):2583‐2596. doi:10.7150/IJBS.71519 35414787 PMC8990466

[35] Luo Y , Xuan Z , Zhu X , Zhan P , Wang Z . Long non‐coding RNAs RP5‐821D11.7, APCDD1L‐AS1 and RP11‐277P12.9 were associated with the prognosis of lung squamous cell carcinoma. Mol Med Rep. 2018;17(5):7238‐7248. doi:10.3892/MMR.2018.8770 29568882 PMC5928681

[36] Wu J , Zheng C , Wang Y , et al. LncRNA APCDD1L‐AS1 induces icotinib resistance by inhibition of EGFR autophagic degradation via the miR‐1322/miR‐1972/miR‐324‐3p‐SIRT5 axis in lung adenocarcinoma. Biomark Res. 2021;9(1):9. doi:10.1186/S40364-021-00262-3 33516270 PMC7847171

[37] Li S , Shi Z , Fu S , et al. Exosomal‐mediated transfer of APCDD1L‐AS1 induces 5‐fluorouracil resistance in oral squamous cell carcinoma via miR‐1224‐5p/nuclear receptor binding SET domain protein 2 (NSD2) axis. Bioengineered. 2021;12(1):7177‐7193. doi:10.1080/21655979.2021.1979442 PMC8806529 34546854

[38] Ma J , Kong FF , Yang D , et al. lncRNA MIR210HG promotes the progression of endometrial cancer by sponging miR‐337‐3p/137 via the HMGA2‐TGF‐β/Wnt pathway. Mol Ther Nucleic Acids. 2021;24:905‐922. doi:10.1016/J.OMTN.2021.04.011 34094710 PMC8141672

[39] Du Y , Wei N , Ma R , et al. Long noncoding RNA MIR210HG promotes the Warburg effect and tumor growth by enhancing HIF‐1α translation in triple‐negative breast cancer. Front. Oncologia. 2020;10:580176. doi:10.3389/FONC.2020.580176 PMC7774020 33392077

[40] Li XY , Zhou LY , Luo H , et al. The long noncoding RNA MIR210HG promotes tumor metastasis by acting as a ceRNA of miR‐1226‐3p to regulate mucin‐1c expression in invasive breast cancer. Aging. 2019;11(15):5646‐5665. doi:10.18632/AGING.102149 31399552 PMC6710038

[41] Shi W , Tang Y , Lu J , Zhuang Y , Wang J . MIR210HG promotes breast cancer progression by IGF2BP1 mediated m6A modification. Cell Biosci. 2022;12(1):38. doi:10.1186/S13578-022-00772-Z 35346372 PMC8962467

[42] Wang AH , Jin CH , Cui GY , et al. MIR210HG promotes cell proliferation and invasion by regulating miR‐503‐5p/TRAF4 axis in cervical cancer. Aging. 2020;12(4):3205‐3217. doi:10.18632/AGING.102799 32087604 PMC7066889

[43] Bu L , Zhang L , Tian M , Zheng Z , Tang H , Yang Q . LncRNA MIR210HG facilitates non‐small cell lung cancer progression through directly regulation of miR‐874/STAT3 Axis. Dose Response. 2020;18(3):155932582091805. doi:10.1177/1559325820918052 PMC7357071 32699535

[44] Kang X , Kong F , Huang K , et al. LncRNA MIR210HG promotes proliferation and invasion of non‐small cell lung cancer by upregulating methylation of CACNA2D2 promoter via binding to DNMT1. Onco Targets Ther. 2019;12:3779‐3790. doi:10.2147/OTT.S189468 31190878 PMC6529604

[45] Li ZY , Xie Y , Deng M , et al. c‐Myc‐activated intronic miR‐210 and lncRNA MIR210HG synergistically promote the metastasis of gastric cancer. Cancer Lett. 2022;526:322‐334. doi:10.1016/J.CANLET.2021.11.006 34767926

[46] Lan H , Zeng J , Chen G , Huang H . Survival prediction of kidney renal papillary cell carcinoma by comprehensive LncRNA characterization. Oncotarget. 2017;8(67):110811‐110829. doi:10.18632/ONCOTARGET.22732 29340018 PMC5762286

[47] Xiao Y , Yu D . Tumor microenvironment as a therapeutic target in cancer. Pharmacol Ther. 2021;221:221. doi:10.1016/J.PHARMTHERA.2020.107753 PMC8084948 33259885

[48] Wang W , Ye Y , Zhang X , Sun W , Bao L . An angiogenesis‐related three‐long non‐coding ribonucleic acid signature predicts the immune landscape and prognosis in hepatocellular carcinoma. Heliyon. 2023;9(3):e13989. doi:10.1016/j.heliyon.2023.e13989 36873490 PMC9982620

