# Supplementary material for: Cervical cancer‐specific long non‐coding RNA landscape reveals the favorable prognosis predictive performance of an ion‐channel‐related signature model
Source: Cancer Med. 2024 Jun 12;13(11):e7389. doi: 10.1002/cam4.7389 (PMC11167610; doi:10.1002/cam4.7389)
Supplement: Supplementary file 1 — Figures S1–S3. [file CAM4-13-e7389-s002.docx]

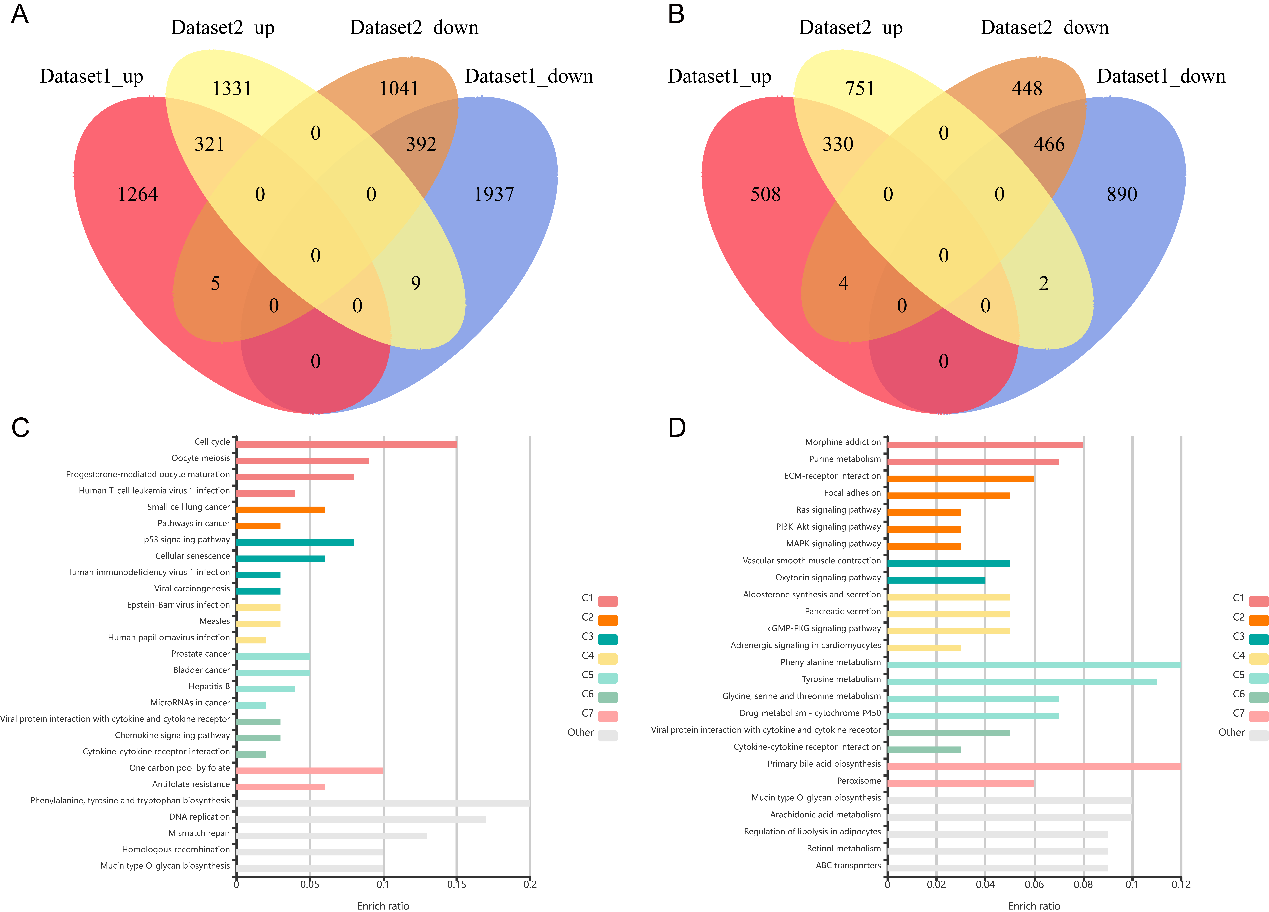


**Supplementary Figure 1.** Identification of TR-lncRNAs by comparing tumor with normal samples in CC. (A, B) Venn plot showed the overlapped differentially expressed lncRNAs (A) and coding genes (B) between the two datasets. (C, D) Bar plots showed the KEGG enrichment pathways of upregulated (C) and downregulated coding genes (D).


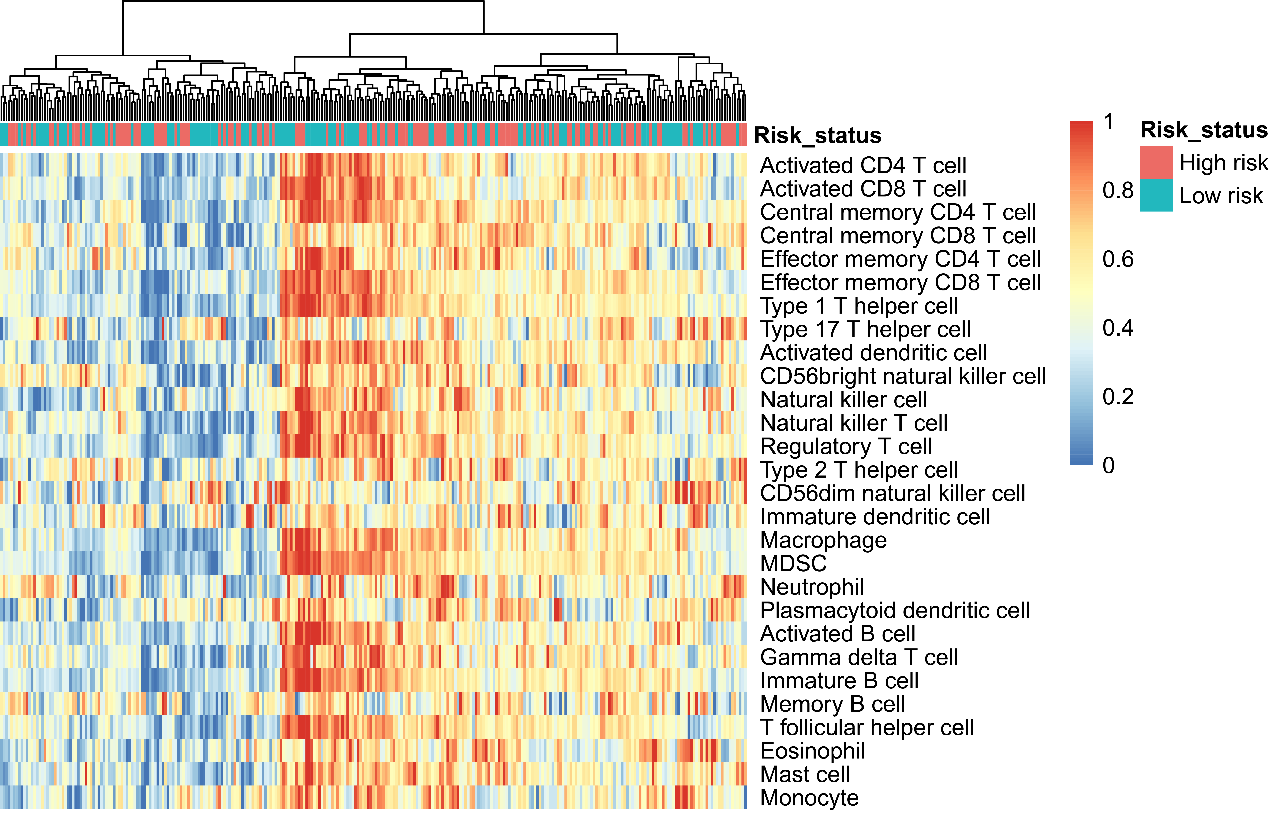


**Supplementary Figure 2.** The infiltration abundance of 28 immune cell types was evaluated by the ssGSEA algorithm.


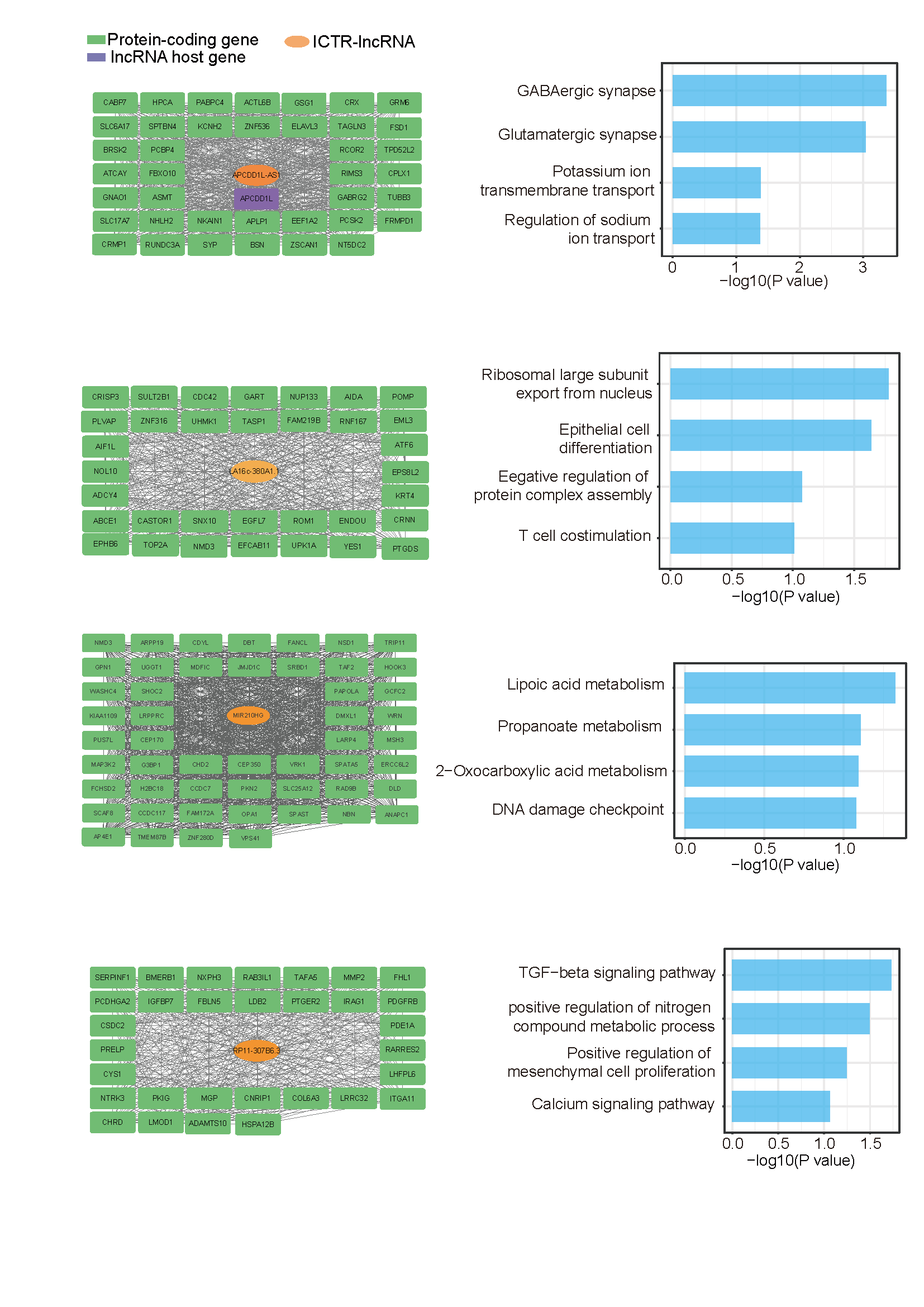

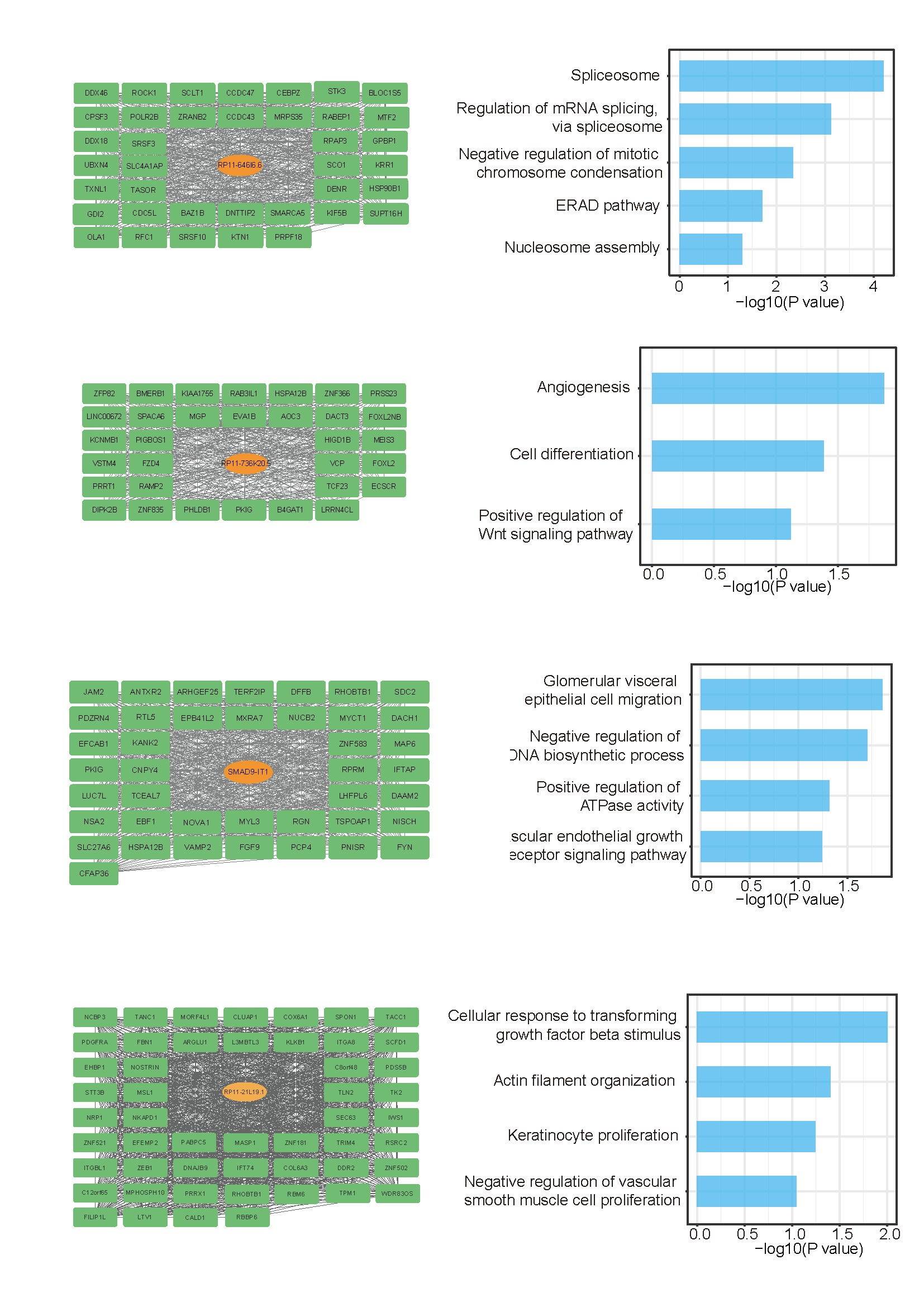


**Supplementary Figure 3.** The co-expression network for ICTR-lncRNA and prediction of related functions. Co-expressed protein-coding genes, lncRNA genes and the host gene of lncRNA genes are colored in green, orange, and purple, respectively.
